# Assessment of predictive validity of the Cognitive Adaptability and Resiliency Employment Screener (CARES) among content moderators

**DOI:** 10.3389/fpsyt.2026.1667014

**Published:** 2026-02-13

**Authors:** John Caesar de Villa, Ashley Edwards, Marlyn Thomas Savio, Jolguer Perez, Xieyining Huang, Rachel Lutz Guevara

**Affiliations:** 1 Wellness & Resiliency Division of Research, TaskUs Inc., New Braunfels, TX, United States; 2 Florida Center for Reading Research, Florida State University, Tallahassee, FL, United States

**Keywords:** assessment, content moderation, hiring, predictive validity, psychometrics

## Abstract

**Introduction:**

Content moderators safeguard the internet from harmful content, and in the process, encounter the risk of traumatic exposure and negative wellbeing outcomes. Protecting their psychological health begins at the recruitment phase. The Cognitive Adaptability and Resiliency Employment Screener (CARES) is one such psychometric instrument that screens for traits and qualities which will aid success in content moderation. Previous research had established the reliability, and convergent and divergent validity of CARES among content moderators in the Philippines. This study investigated the predictive validity of CARES in respect of critical psychological outcomes–secondary traumatic stress, burnout, compassion satisfaction, perceived stress, and resilience.

**Method:**

A sample of 336 content moderators in the Philippines who completed CARES during the hiring stage were given wellness surveys (at 6-month intervals) to assess for psychological outcomes across time.

**Results:**

Findings based on linear mixed effect models showed that CARES was able to significantly predict different wellness measures with effect sizes ranging between -0.33 and 0.38. Furthermore, the interaction between CARES dimensions and time across most models was not significant, indicating that the predictive capability of CARES did not significantly vary across time.

**Discussion:**

The results indicated that CARES has sound predictive validity for psychological health outcomes relevant to content moderators.

## 1 Introduction

Content moderators are professionals who review user generated material on digital platforms to ensure policy compliance and user safety (1). Given the wide range of heinous content online (e.g., nudity, misinformation, violence, child abuse, and gore), moderators encounter the potential risk of occupational injury (e.g., secondary traumatic stress, burnout) like most frontline workers such as medical, armed forces and emergency personnel (2). Employers therefore have a duty of care to protect content moderators from such hazards and ensure their safety in the long run. A critical first step is to ensure that candidate fitness is thoroughly evaluated for content moderation work. The Cognitive Adaptability and Resiliency Employment Screener (CARES) is a recent psychometrically validated employment screener developed with special focus on gauging the cognitive and psychological qualities essential for content moderation jobs (3).

To the best of our knowledge, no other psychometric screening instrument besides CARES currently exists in the public domain for evaluating content moderation candidates. In the original empirical report (3), CARES was tested in a sample of over 4,800 full-time content moderators in the Philippines (which is a popular outsourcing shore for moderation) across three phases. While the first two phases revealed a 3-factor structure (Psychological Perseverance & Agility, Rumination & Emotional Lingering, and Expressiveness & Sociability), the third phase found the tool’s factors to have excellent internal consistency (Cronbach’s alpha ranged between 0.77 and 0.96, McDonald’s omega ranged between 0.87 and 0.97). However, the factors had moderate average variance extracted (highest being 0.39 as against the recommended 0.50 or more), which posits the need to look at the scale in entirety in future investigations. Nonetheless, CARES factors had demonstrated adequate convergent and divergent validity with other established psychometric scales for resilience, cognitive control and flexibility, optimism-pessimism, worry, and emotion regulation. Evidence from the preliminary study thus presents CARES as a viable instrument for real-world screening purposes to recruit the best fit candidates who can flourish on the job.

Psychometric tools such as CARES are especially useful for assessing latent constructs (e.g., resilience for content moderation work) that otherwise are difficult to measure and articulate. To understand why CARES may be relevant for longitudinal success in content moderation type work, it is useful to examine the daily flow, tasks and schedule of moderators. In a typical shift, content moderators parse through hundreds of flagged digital material in various formats (images, video, audio, text) with specific policies and guidelines in mind to determine violations and take corresponding actions. This fundamental task requires handling volumes and diverse types of content with close attention to details and nuances on a global scale. Job postings for the role explicitly seek empathy, cultural awareness, flexibility, and analytical and decisional skills besides technical requirements based on the line of work (4). Yet, these latent socioaffective and cognitive qualities are not often systematically assessed in the screening process, leaving a key gap in choosing the best fits for the role, and potentially leading to distress, conflicts and attrition in moderation teams (5, 6).

CARES was found to have three dimensions with adequate initial reliability and validity—psychological perseverance and agility (PPA), rumination and emotional lingering (REL), and expressiveness and sociability (ESc) (3). PPA (ability to psychologically adapt and regulate oneself) and REL (rigidity with emotional and cognitive shifting) together probe emotional control, problem solving, optimism, flexibility and tenacity. The theme underlying these abilities is resilience (defined as the cognitive and behavioral efforts to protect and promote one’s wellbeing in the face of stressors) (7). With increasing attention being drawn to psychological risks for frontline workers, organizations have implemented resilience-themed and trauma-informed programs (8), and hence it is arguable that screening a candidate’s potential for resilience-related constructs may improve their gains in future training and outcomes in the long run.

The ESc dimension sheds light on the importance of social expressivity in relation to one’s emotional health. Past literature emphasizes that moderators rely extensively on team relations and support in coping with daily challenges and finding a frame of reference or normalization in a job that is less understood or cannot be discussed much beyond the workplace (9, 10). The emotional labor inherent in content moderation work can potentiate emotional suppression and eventually exhaustion especially given the engagement with unconventional and explicit content. The ESc dimension offers information on prospective employees’ emotional expressivity. Put together, with PPA and ESc representing protective factors, and REL indicating risk in the content moderation context, CARES offers a balanced screening method as well as a guide for training needs.

Resilience, which tends to be the central theme of training programs, has long been contended in the state vs trait debate (11). Yet, the American Psychological Association in 2020 clarified that resilience is common to all humans and manifests in a combination of learned behaviors, thoughts and actions for overcoming challenges (12). The 2024 issue of *Frontiers in Psychology* on Emotional Resilience for Wellbeing and Employability: The Role of Learning and Training highlighted that although across professions, the demands and resources vary, resilience is trainable effectively when relevant emotional models and principles are considered in program development (13). This reiterates the advantage of having an instrument like CARES that identifies moderator-specific competencies which play a role in their wellbeing, and can therefore support training in a more tailored manner. There is, nonetheless, a need to explore further how well the tool corresponds to longitudinal wellbeing outcomes among content moderators.

Some key psychometric indices are commonly relied on by users of the tool to get a quick assessment of the instrument’s relevance and utility (14). These include reliability and validity. While in the early phases of tool construction, preliminary reliability and validity are prioritized, it is important to revisit and evaluate the real world usefulness of the tool, i.e., how well the tool can forecast outcomes of related phenomena (also known as criterion). Predictive validity comes handy in this regard. It is a type of criterion validity which helps establish the predictive relationship between the tool and a criterion (e.g., the extent to which an intelligence test can predict academic performance) (15). This predictive power arguably helps provide support for the utility of the construct being measured by demonstrating that it is a critical factor at play in long-term outcomes.

For a niche high-risk population like content moderators, it is extremely important to have tools that can predict their long-term success and adversities. The nature of work in content moderation necessitates a futuristic care focus so that occupational risks are preempted and mitigated. For example, moderators require a period of time (say 6 months) to habituate to their work and begin showing success (16). It is apparent then that simply screening candidates while hiring without any evidence that such assessment holds value in the long run is futile. A psychometric instrument must demonstrate the ability to accurately predict the outcomes of interest that are intended to be screened for.

An important next step for CARES is to exhibit the ability to gauge future outcomes. This will further empower employers to take adequate and specific steps to safeguard moderators against risks on the job. It holds the added potential benefit for employers to comply with labor regulations by extending preventive measures from the outset. The current study, therefore, attempts to investigate the predictive validity of CARES in terms of employee wellbeing, measured by existing psychometric tools. We include positive and negative criterion variables of interest in the content moderator population, as discussed by past literature (2, 17)—resilience and risks (burnout, secondary traumatic stress, compassion satisfaction).

## 2 Method

The study methods were approved by the ethics review board at TaskUs Inc., which was chaired by an independent external mental health researcher at a major US university, and were in compliance with the employee code of conduct and legal regulations stipulated by TaskUs Inc. There were no adverse outcomes reported as a result of participation in this research.

### 2.1 Participants and procedure

The study sample consisted of candidates participating in the hiring process for content moderator roles at TaskUs in the Philippines. Inclusion criteria required participants to: (a) be at least 18 years old; (b) complete CARES and biannual wellness surveys conducted by the organization; and (c) consent to research involving matching of these survey data. Baseline procedures were previously described in detail by Torralba et al. (3). In brief, all candidates applying to content moderator roles at TaskUs were invited to voluntarily complete CARES for research purposes between December 2021 and October 2023. They were informed that their participation or non-participation would not impact their recruitment outcome. The data were accessible only to the researchers who were part of an independent team in the organization with no oversight or management of candidates or employees.

Candidates who were hired and successfully onboarded as full time content moderators and had completed biannual wellness assessments (meant for all TaskUs employees) were considered for the current study. The sample consisted of 336 content moderators who consented for their CARES data to be matched with biannual data. Within this sample, there was a near even gender split (48% male, 47% female, 5% other). In terms of age distribution, the majority were within the 25–30 years age group (38%), followed by 31–35 years (20%), 18–24 years (17%), 36–40 years (13%), 41–50 years (11%), 51–60 years (1%) and undisclosed (1%).

To assess the effect of time in the model, timepoints were derived at 6-month intervals from the start of the job. The 6-month interval between surveys has been a standard practice in the organization to ensure periodic data gathering while avoiding the risk of survey fatigue. Given that the wellness survey involved voluntary participation, employees at the time of the study had the option to skip participating in a particular wellness survey period. This resulted in missing data at different points. Hence, the model only analyzed whatever available periods each employee had. Timepoint 0 (T0) contained wellness survey results from 336 content moderators who were in the company in their first 0–6 months (M = 5.99, SD = 0.28). Timepoint 1 (T1) had wellness data from 331 content moderators who were in the company with 7–12 months’ tenure (M = 11.89, SD = 0.70). Timepoint 2 (T2) covered data from 320 employees with 13–18 months’ tenure (M = 17.9, SD = 0.67). At Timepoint 3 (T3), 309 employees with 19–24 months’ tenure were included (M = 23.73, SD = 1.05). For timepoint 4 (T4), there were 283 employees with 25–30 months’ tenure (M = 29.89, SD = 0.73). Timepoint 5 (T5) had 272 content moderators with tenure of 31–36 months (M = 35.89, SD = 0.69). Timepoint 6 (T6) contained 262 employees’ data whose tenure was in the range of 37–42 months (M = 42.00, SD = 0.12). Timepoint 7 (T7) consisted of 261 moderators having a tenure of 43–48 months (M = 47.98, SD = 0.28). At timepoint 8 (T8), there were 260 content moderators tenured 49–54 months (M = 53.98, SD = 0.38). Lastly, Timepoint 9 (T9) contained 258 employees between 55 and 60 months’ tenure (M = 59.98, SD = 0.25).

### 2.2 Measures

#### 2.2.1 Cognitive adaptability and resilience scale

CARES is a 75-item employment screening tool designed to gauge psychosocial and cognitive traits important for content moderation work (3). Each item is rated on a 7-point Likert scale from 0 (“Strongly Disagree”) to 6 (“Strongly Agree”). CARES comprises three subscales: Psychological Perseverance & Agility (PPA; e.g. I tend to think more logically instead of emotionally, I am able to remain calm following any stressful situation), Rumination & Emotional Lingering (REL; e.g. I get distracted by my thoughts, I become easily overwhelmed when provided too many tasks), and Expressiveness & Sociability (ESc; e.g. I would consider myself as someone who shares feelings openly). In the present sample, CARES had high internal consistency for PPA (Cronbach’s alpha = 0.90), and REL (Cronbach’s alpha = 0.93) while an acceptable internal consistency for ESc (Cronbach’s alpha = 0.77).

#### 2.2.2 Connor Davidson resilience scale 10

The CD-RISC 10 is a 10-item scale that measures resilience or how well equipped a person is to bounce back after stressful events, tragedy, or trauma (18). It contains items such as “I am able to adapt when changes occur”, rated on a 5-point Likert scale ranging between 0 (“Not True at All”) to 4 (“True Nearly All the Time”). The scale score ranges between 0 and 40, with 0–29 considered low resilience, 30–36 being moderate/intermediate resilience, and 37–40 indicating high resilience (23). The scale showed good internal consistency (Cronbach’s alpha is between 0.85 to 0.91 across the study period).

#### 2.2.3 Professional quality of life

ProQOL, consisting of 30 items, measures how one feels about their work as a helper (19). It contains three subscales: Compassion Satisfaction, Burnout, and Secondary Traumatic Stress. Sample items include, “I feel invigorated after working with those I help.”, “Because of my helping, I have felt “on edge” about various things.” Items are rated on a 5-point Likert scale ranging between 1 (“Never”) and 5 (“Very Often”). The score for each subscale ranges between 10 and 50, and is interpreted as follows—between 10 and 22 signifies low range, between 23 and 41 represents average/moderate range, and above 42 suggests high range. ProQOL demonstrated excellent internal consistency (Cronbach’s alpha= 0.95) in the current sample.

#### 2.2.4 Perceived stress scale

The 10-item PSS evaluates the extent to which the respondent considers life situations to be stressful. It provides the respondent’s perception of stress during the preceding month (20). Items cover general aspects of life such as, “In the last month, how often have you felt confident about your ability to handle your personal problems?”, “In the last month, how often have you found that you could not cope with all the things that you had to do?”. Each item is rated on a 5-point Likert scale between 0 (“Never”) and 4 (“Very Often”). The overall score falls in the 0–40 range such that 0–13 indicates low stress, 14–26 suggests moderate stress, and above 27 represents high stress. PSS exhibited good internal consistency (Cronbach’s alpha is between 0.88 to 0.90 across the study period) in the current sample.

### 2.3 Statistical analysis

Linear mixed effects models were used to explore the relation between CARES and psychological outcomes, with a random intercept included for persons to account for the nested structure of the data with repeated measures nested within individuals. Random slopes for time were not included because most participants contributed data at only 2–3 of the 10 timepoints, providing insufficient information to reliably estimate individual time slopes, thus time was treated as a fixed effect. Time was treated as a continuous variable in this with Time 1 coded as 1, and so on. Linear slopes were used after the exploratory inspection using spaghetti plots did not demonstrate any clear non-linear patterns that would justify the addition of any quadratic terms. Time was z-scored like all of the other variables in the model to aid in interpretation such that 1 represents 1SD above the mean. Models were fit in R using the lme4 package (21) with a random intercept for person. A series of models were conducted for each of the psychological outcomes of interest (Burnout, Compassion Satisfaction, Secondary Traumatic Stress, Resilience, and Perceived Stress). First, an unconditional model was run to determine the amount of variance between individuals (random intercept variance) and within individuals across time (residual variance). Next, individual models were run with each predictor as the only predictor included to evaluate the amount of variance in the outcome explained by that predictor alone. Third, we ran a simultaneous model with all predictors entered simultaneously to explore the amount of variance that can be explained by all predictors together as well as to see which predictors explain unique variance unaccounted for by the other predictors. Lastly, an interaction model was conducted with timepoint as the moderator to determine whether the relation between the prescreen measures and the psychological outcome varies across time. All variables were z-scored to aid in interpretation of coefficients such that coefficients represent the number of standard deviation unit changes in the outcome observed for a 1 standard deviation change in the predictor.

Amount of variance explained was evaluated to see how much of the variance in person intercepts can be accounted for by the predictors. The proportion of between-person variance explained by the predictors was quantified using a level-2 proportional reduction in variance (*R^2^*), calculated as the reduction in random-intercept variance from the unconditional model to the conditional model divided by the random-intercept variance in the unconditional model.

Models were conducted using Restricted Maximum Likelihood (REML) as it provides unbiased estimators and takes into account the loss of degrees of freedom associated with estimating fixed effects, leading to less biased estimates of variance components. REML is more robust for cases where we cannot assume data are missing at random. Little’s MCAR test revealed data to not be missing completely at random (*χ*^2^(1833) = 3413, *p* < 0.001) and given that survey completion was voluntary, it is possible that the presence of missing data could relate to the true value. Also, because responses were missing due to voluntary participation, estimates from the linear mixed‐effects model rely on an assumption that missingness is conditionally missing at random given the observed data. Under this assumption, the screening measure can be interpreted as predicting individual differences in the outcome. However, if missingness depends on unobserved outcome values (i.e., is missing not at random), the estimated association should be interpreted more conservatively as a conditional association among observed responses rather than as an unbiased estimate of the screening measure’s predictive relationship in the full target population. Given all of this, REML provides a robust framework for reducing bias while utilizing the repeated measures where available in the presence of large missing data patterns.

#### 2.3.1 Predictor variables

The three CARES dimensions–Psychological Perseverance & Agility (PPA), Rumination & Emotional Lingering (REL), and Expressiveness & Sociability (ESc)–derived from the results of the previous study (3) were utilized to evaluate the predictive validity of longitudinal outcomes. In addition, time was also included as a predictor to assess how time influenced the outcomes.

#### 2.3.2 Outcome variables

Scores across different timepoints were considered for Burnout, Compassion Satisfaction, Secondary Traumatic Stress, Resilience, and Perceived Stress. These values are continuous in nature, and hence fit for a linear mixed effects model analysis.

Information on the model fit and selection of the CARES scale can be found in the original publication (3).

## 3 Results

### 3.1 Predictive validity

**Table 1** contains bivariate correlations on the lower triangle and the number of participants with available data for that correlation on the upper triangle. Data were available from all 336 participants on the CARES dimensions (PPA, REL, and ESc), whereas psychological outcomes during the time of employment contain missing data based on what time periods participants completed the voluntary biannual survey for employment time at the time of the surveys. Note that perceived stress was added to the survey in more recent years resulting in a smaller sample size for that outcome based on tenure at the time in which that occurred in the survey (rather than participants choosing not to respond specifically to that scale).

**Table 1:** Pearson correlations between and among screening measures and outcomes at the first timepoint.

	PPA	REL	ESc	Burnout	Compassion satisfaction	Secondary traumatic stress	Resilience	Perceived stress
PPA	336	336	336	190	181	179	140	71
REL	-0.66***	336	336	190	181	179	140	71
ESc	0.28***	-0.16**	336	190	181	179	140	71
Burnout	-0.24***	0.31***	-0.11	190	113	94	58	22
Compassion Satisfaction	0.41***	-0.36***	0.18*	-0.68***	181	111	74	26
Secondary Traumatic Stress	-0.05	0.23**	0.06	0.7***	-0.35***	179	100	37
Resilience	0.42***	-0.36***	0.08	-0.52***	0.64***	-0.37***	140	51
Perceived Stress	-0.25*	0.31**	0.04	0.58***	-0.5***	0.41***	-0.52***	71

The lower triangle presents correlations with significance level and the upper triangle presents the pairwise sample size. *p<.05, **p<.01, ***p<.001. PPA, Psychological Perseverance and Agility; REL, Rumination and Emotional Lingering; ESc, Expressiveness and Sociability.

Results showed a moderate negative correlation between PPA and REL such that individuals who scored high on PPA were more likely to score low on REL. Small yet significant correlations were also observed between PPA and REL with ESc. High correlation was observed between burnout with secondary traumatic stress (*r* = 0.70) and compassion satisfaction (*r* = -0.68). This may be an indication of some conceptual overlap between the two metrics. However, this does not impact subsequent models as these metrics were modeled separately as dependent variables. This eliminates the assumption of multicollinearity which affects the validity of the results. Nonetheless, caution is necessary when reading the tables.

**Table 2** presents the descriptive statistics for the number of available datapoints for each individual and the average of those scores across time. All 336 participants had burnout data, between 1 and 7 timepoints available. Of these 336 participants, the average number of available timepoints was 2.64 with a standard deviation of 1.24. Similar patterns were observed across the other psychometric measures except for Perceived Stress having fewer timepoints on average which reflects the fact that this scale was added to the survey later and thus was only available for fewer timepoints.

**Table 2:** Descriptives for wellbeing measures: group level mean of average individual-level scores from across timepoints (n=336).

	Mean of average score	SD of average score	Mean number of timepoints	SD of number of timepoints
Burnout	17.84	5.29	2.64	1.24
Compassion Satisfaction	44.08	5.04	2.64	1.24
Secondary Traumatic Stress	20.07	6.51	2.57	1.22
Resilience	34.92	4.5	2.6	1.25
Perceived Stress	14	7.8	2.12	0.89

Average scores were calculated as a person’s average score across all timepoints for which data were available. Mean of average represents the group level mean of those average within person scores.

#### 3.1.1 Burnout

**Table 3** presents results of the linear mixed effects models predicting burnout. Results of the individual models with each predictor entered separately revealed that both PPA and REL were significant predictors of Burnout, whereas time and ESc did not demonstrate a significant relation with burnout. PPA had a negative association with burnout such that an individual who scored 1SD higher on PPA measure, was 0.21SD less burnt out. Whereas REL showed a positive association with burnout such that for a 1SD increase in REL, individuals scored 0.31 higher on burnout. When all predictors were entered into the model simultaneously, REL was the only significant predictor suggesting that REL contributes unique information unable to be accounted for by the other predictors. With all predictors entered simultaneously, these predictors explained 17% of the individual differences in burnout. Lastly, the interaction model revealed no significant interaction with time.

**Table 3:** Linear mixed effects models predicting burnout.

	Estimate	Standard error (SE)	95% CI	P-value	Random effects		Model fit	
					Residual variance	Person intercept variance	AIC	BIC
Model 1: Unconditional model								
(Intercept)	0.01	0.05	-0.09 – 0.10	0.916	0.43	0.57	2257.82	2272.19
Model 2: Individual time model								
(Intercept)	0.00	0.06	-0.12 – 0.11	0.973	0.43	0.57	2264.31	2283.47
time	-0.01	0.04	-0.09 – 0.07	0.840				
Model 3: Individual PPA model								
(Intercept)	0.01	0.05	-0.08 – 0.10	0.855	0.43	0.52	2243.45	2262.6
PPA	-0.21	0.05	-0.30 – -0.12	**<0.001**				
Model 4: Individual REL model								
(Intercept)	0.01	0.04	-0.08 – 0.10	0.848	0.43	0.47	2220.35	2239.5
REL	0.31	0.04	0.22 – 0.39	**<0.001**				
Model 5: Individual ESc model								
(Intercept)	0.01	0.05	-0.09 – 0.10	0.895	0.43	0.56	2260.41	2279.57
ESc	-0.09	0.05	-0.18 – 0.00	0.055				
Model 6: Simultaneous model								
(Intercept)	0.02	0.06	-0.10 – 0.13	0.788	0.43	0.47	2238.12	2271.65
time	0.01	0.04	-0.07 – 0.09	0.862				
PPA	-0.01	0.06	-0.13 – 0.11	0.885				
REL	0.29	0.06	0.18 – 0.41	**<0.001**				
ESc	-0.04	0.05	-0.13 – 0.05	0.382				
Model 7: Interaction model								
(Intercept)	0.01	0.06	-0.10 – 0.13	0.803	0.43	0.47	2255.78	2303.67
time	0.01	0.04	-0.08 – 0.09	0.88				
PPA	0.05	0.08	-0.12 – 0.21	0.561				
REL	0.34	0.08	0.18 – 0.50	**<0.001**				
ESc	-0.05	0.06	-0.17 – 0.07	0.395				
time × PPA	0.06	0.06	-0.06 – 0.19	0.319				
time × REL	0.05	0.06	-0.07 – 0.18	0.371				
time × ESc	-0.01	0.04	-0.10 – 0.07	0.762				

Model coefficients can be interpreted as standardized effect sizes given that all variables were z-scored. P-values representing significance at p<.05 are bolded. PPA, Psychological Perseverance and Agility; REL, Rumination and Emotional Lingering; ESc, Expressiveness and Sociability.

#### 3.1.2 Compassion satisfaction

Results of the models predicting compassion satisfaction can be found in **Table 4**. In the individual models, all three CARES dimensions were significant predictors of compassion satisfaction whereas time was not. Both PPA and ESc were found to be positively associated with compassion satisfaction such that higher scores on these were related to higher compassion satisfaction scores. Whereas REL had a negative association with compassion satisfaction such that those who scored 1SD higher on the REL prescreen measure had 0.33SD lower compassion satisfaction. In the simultaneous predictor model, both PPA and REL were found to provide significant unique contributions to the prediction of compassion satisfaction whereas neither ESc or time explain significant variance above what could be accounted for by the other predictors. Together these predictors explained 24% of the individual differences in compassion satisfaction. Lastly, the interaction model revealed no significant interaction with time.

**Table 4:** Linear mixed effects models predicting compassion satisfaction.

	Estimate	Standard error (SE)	95% CI	P-value	Random effects		Model fit	
					Residual variance	Person intercept variance	AIC	BIC
Model 1: Unconditional model								
(Intercept)	0	0.05	-0.09 – 0.09	0.957	0.44	0.54	2269.17	2283.53
Model 2: Individual time model								
(Intercept)	-0.04	0.06	-0.15 – 0.08	0.54	0.44	0.54	2274.78	2293.93
time	-0.04	0.04	-0.12 – 0.04	0.341				
Model 3: Individual PPA model								
(Intercept)	-0.01	0.04	-0.09 – 0.08	0.848	0.44	0.44	2227.2	2246.35
PPA	0.32	0.04	0.23 – 0.40	**<0.001**				
Model 4: Individual REL model								
(Intercept)	-0.01	0.04	-0.09 – 0.08	0.871	0.44	0.44	2220.99	2240.14
REL	-0.33	0.04	-0.42 – -0.25	**<0.001**				
Model 5: Individual ESc model								
(Intercept)	0	0.05	-0.10 – 0.09	0.92	0.44	0.52	2266.42	2285.58
ESc	0.14	0.05	0.05 – 0.23	**0.002**				
Model 6: Simultaneous model								
(Intercept)	-0.06	0.05	-0.16 – 0.05	0.288	0.44	0.41	2227.36	2260.88
time	-0.06	0.04	-0.14 – 0.02	0.154				
PPA	0.15	0.06	0.04 – 0.27	**0.01**				
REL	-0.22	0.06	-0.33 – -0.11	**<0.001**				
ESc	0.07	0.04	-0.02 – 0.15	0.136				
Model 7: Interaction model								
(Intercept)	-0.05	0.06	-0.15 – 0.06	0.404	0.45	0.41	2242.8	2290.69
time	-0.05	0.04	-0.13 – 0.03	0.257				
PPA	0.07	0.08	-0.09 – 0.23	0.385				
REL	-0.25	0.08	-0.40 – -0.09	**0.002**				
ESc	0.07	0.06	-0.05 – 0.18	0.261				
time × PPA	-0.09	0.06	-0.22 – 0.03	0.153				
time × REL	-0.02	0.06	-0.14 – 0.10	0.706				
time × ESc	0	0.04	-0.09 – 0.08	0.976				

Model coefficients can be interpreted as standardized effect sizes given that all variables were z-scored. P-values representing significance at p<.05 are bolded. PPA = Psychological Perseverance and Agility, REL, Rumination and Emotional Lingering; ESc, Expressiveness and Sociability.

#### 3.1.3 Secondary traumatic stress

Results of the prediction of secondary traumatic stress are presented in **Table 5**. Individual predictor models showed that secondary traumatic stress was significantly predicted by time, PPA and REL, but not ESc. Time was found to be negatively associated with secondary traumatic stress such that individuals reported lower secondary traumatic stress the longer that they had been working for the company. PPA was found to be negatively associated with secondary traumatic stress such that those with higher PPA on CARES reported lower secondary traumatic stress on average. Whereas a positive association was observed with REL such that higher REL was associated with higher secondary traumatic stress. When predictors were entered simultaneously, both time and REL were found to significantly predict secondary traumatic stress, with this simultaneous prediction explaining 10% of the variance in secondary traumatic stress between individuals. However, there was no interaction with time meaning that individuals simply reported lower secondary traumatic stress the longer they had been with the company but this did not change based on CARES dimensions.

**Table 5:** Linear mixed effects models predicting secondary traumatic stress.

	Estimate	Standard error (SE)	95% CI	P-value	Random effects		Model fit	
					Residual variance	Person intercept variance	AIC	BIC
Model 1: Unconditional model								
(Intercept)	0.01	0.05	-0.08 – 0.10	0.822	0.5	0.49	2267.51	2281.79
Model 2: Individual time model								
(Intercept)	-0.08	0.06	-0.20 – 0.03	0.169	0.49	0.51	2267.71	2286.76
time	-0.11	0.04	-0.20 – -0.02	**0.012**				
Model 3: Individual PPA model								
(Intercept)	0.01	0.05	-0.08 – 0.10	0.779	0.5	0.48	2267.55	2286.6
PPA	-0.12	0.05	-0.21 – -0.03	**0.012**				
Model 4: Individual REL model								
(Intercept)	0.01	0.04	-0.07 – 0.10	0.749	0.5	0.43	2244.83	2263.87
REL	0.24	0.04	0.16 – 0.33	**<0.001**				
Model 5: Individual ESc model								
(Intercept)	0.01	0.05	-0.08 – 0.10	0.833	0.5	0.49	2273.29	2292.34
ESc	0.03	0.05	-0.06 – 0.12	0.467				
Model 6: Simultaneous model								
(Intercept)	-0.07	0.06	-0.19 – 0.04	0.194	0.49	0.44	2253.53	2286.86
time	-0.1	0.04	-0.19 – -0.02	**0.016**				
PPA	0.07	0.06	-0.05 – 0.19	0.28				
REL	0.3	0.06	0.18 – 0.41	**<0.001**				
ESc	0.07	0.05	-0.02 – 0.17	0.105				
Model 7: Interaction model								
(Intercept)	-0.07	0.06	-0.18 – 0.05	0.254	0.49	0.44	2270.2	2317.81
time	-0.1	0.04	-0.18 – -0.01	**0.029**				
PPA	0.07	0.09	-0.10 – 0.24	0.403				
REL	0.34	0.08	0.18 – 0.51	**<0.001**				
ESc	0.07	0.06	-0.05 – 0.18	0.285				
time × PPA	0	0.07	-0.13 – 0.14	0.943				
time × REL	0.06	0.06	-0.07 – 0.18	0.391				
time × ESc	-0.01	0.05	-0.10 – 0.08	0.773				

Model coefficients can be interpreted as standardized effect sizes given that all variables were z-scored. P-values representing significance at p<.05 are bolded. PPA; Psychological Perseverance and Agility; REL, Rumination and Emotional Lingering; ESc, Expressiveness and Sociability.

#### 3.1.4 Resilience

Results of models predicting resilience can be found in **Table 6**. As individual predictors, all three CARES dimensions were found to be significant predictors of resilience, whereas time was not. PPA and ESc were found to be positively associated with resilience whereas REL was found to have a negative association. When predictors were entered simultaneously, both PPA and REL provided unique contributions to the prediction of resilience whereas time and ESc did not. All of the predictors together explained 28.6% of the individual differences in resilience. Again, no significant interactions with time were observed.

**Table 6:** Linear mixed effects models predicting resilience.

	Estimate	Standard error (SE)	95% CI	P-value	Random effects		Model fit	
					Residual variance	Person intercept variance	AIC	BIC
Model 1: Unconditional model								
(Intercept)	0	0.05	-0.09 – 0.09	0.994	0.47	0.54	2275.59	2289.92
Model 2: Individual time model								
(Intercept)	0.04	0.06	-0.08 – 0.16	0.492	0.47	0.54	2280.79	2299.89
time	0.05	0.04	-0.04 – 0.13	0.258				
Model 3: Individual PPA model								
(Intercept)	-0.01	0.04	-0.09 – 0.07	0.826	0.47	0.39	2211.18	2230.28
PPA	0.38	0.04	0.30 – 0.46	**<0.001**				
Model 4: Individual REL model								
(Intercept)	-0.01	0.04	-0.09 – 0.08	0.896	0.47	0.43	2230.94	2250.04
REL	-0.33	0.04	-0.41 – -0.24	**<0.001**				
Model 5: Individual ESc model								
(Intercept)	0	0.05	-0.09 – 0.09	0.971	0.47	0.52	2275.41	2294.51
ESc	0.12	0.05	0.03 – 0.21	**0.011**				
Model 6: Simultaneous model								
(Intercept)	0.02	0.05	-0.09 – 0.12	0.741	0.47	0.38	2223.52	2256.94
time	0.03	0.04	-0.05 – 0.11	0.415				
PPA	0.28	0.06	0.17 – 0.40	**<0.001**				
REL	-0.13	0.06	-0.24 – -0.02	**0.018**				
ESc	0.02	0.04	-0.07 – 0.11	0.635				
Model 7: Interaction model								
(Intercept)	0.01	0.06	-0.10 – 0.12	0.888	0.47	0.38	2237.98	2285.72
time	0.03	0.04	-0.06 – 0.11	0.544				
PPA	0.24	0.08	0.08 – 0.40	**0.004**				
REL	-0.13	0.08	-0.29 – 0.02	0.093				
ESc	0.09	0.06	-0.02 – 0.21	0.104				
time × PPA	-0.05	0.06	-0.18 – 0.07	0.417				
time × REL	0	0.06	-0.12 – 0.12	0.991				
time × ESc	0.08	0.04	-0.00 – 0.17	0.054				

Model coefficients can be interpreted as standardized effect sizes given that all variables were z-scored. P-values representing significance at p<.05 are bolded. PPA; Psychological Perseverance and Agility, REL; Rumination and Emotional Lingering; ESc, Expressiveness and Sociability.

#### 3.1.5 Perceived stress

**Table 7** provides the results of the models predicting perceived stress. Results of the individual models revealed PPA and REL to significantly predict perceived stress whereas time and ESc did not. However, when entered simultaneously only REL explained unique variance in perceived stress unaccounted for by the other predictors. Again no interaction with time was observed. Together the CARES measures explained 14.4% of the individual differences in perceived stress.

**Table 7:** Linear mixed effects models predicting perceived stress.

	Estimate	Standard error (SE)	95% CI	P-value	Random effects		Model fit	
					Residual variance	Person intercept variance	AIC	BIC
Model 1: Unconditional model								
(Intercept)	0.02	0.05	-0.08 – 0.12	0.705	0.38	0.64	1810.25	1823.92
Model 2: Individual time model								
(Intercept)	0.03	0.06	-0.10 – 0.15	0.689	0.38	0.64	1816.15	1834.39
time	0.01	0.06	-0.10 – 0.12	0.866				
Model 3: Individual PPA model								
(Intercept)	0.02	0.05	-0.08 – 0.12	0.684	0.38	0.6	1803.96	1822.19
PPA	-0.18	0.05	-0.27 – -0.08	**<0.001**				
Model 4: Individual REL Model								
(Intercept)	0.02	0.05	-0.07 – 0.11	0.695	0.38	0.54	1777.66	1795.9
REL	0.3	0.05	0.21 – 0.39	**<0.001**				
Model 5: Individual ESc model								
(Intercept)	0.02	0.05	-0.08 – 0.12	0.706	0.38	0.64	1816.38	1834.62
ESc	0.01	0.05	-0.09 – 0.10	0.918				
Model 6: Simultaneous model								
(Intercept)	0.03	0.06	-0.09 – 0.15	0.631	0.38	0.54	1793.73	1825.64
time	0.02	0.05	-0.09 – 0.12	0.759				
PPA	0.03	0.06	-0.10 – 0.16	0.647				
REL	0.33	0.06	0.21 – 0.46	**<0.001**				
ESc	0.05	0.05	-0.05 – 0.15	0.293				
Model 7: Interaction model								
(Intercept)	0.03	0.06	-0.09 – 0.15	0.635	0.38	0.55	1808.67	1854.25
time	0.02	0.05	-0.09 – 0.12	0.757				
PPA	0.11	0.09	-0.08 – 0.29	0.249				
REL	0.36	0.09	0.18 – 0.53	**<0.001**				
ESc	0.02	0.07	-0.11 – 0.15	0.749				
time × PPA	0.1	0.08	-0.06 – 0.26	0.232				
time × REL	0.03	0.08	-0.13 – 0.19	0.705				
time × ESc	-0.04	0.06	-0.16 – 0.08	0.497				

Model coefficients can be interpreted as standardized effect sizes given that all variables were z-scored. P-values representing significance at p<.05 are bolded. PPA; Psychological Perseverance and Agility; REL, Rumination and Emotional Lingering; ESc, Expressiveness and Sociability.

## 4 Discussion

The main goal of this study was to assess the predictive capability of CARES as a prehire screener for content moderators. Results revealed that CARES was able to significantly predict the following psychological outcomes: burnout, secondary traumatic stress, compassion satisfaction, resilience, and perceived stress, with some dimensions of CARES found to have stronger predictive ability over other dimensions. This underlines the usefulness of CARES in the process of efficiently identifying best fit candidates for content moderation work who are likely to have optimal psychological health outcomes.

Results from linear mixed effects models showed that CARES predicted burnout, secondary traumatic stress, and compassion fatigue which are the major known risks in content moderation work (1, 2). The downstream psychosocial consequences of burnout, secondary traumatic stress and compassion fatigue, as reported in other longstanding helping professions such as nursing and counseling, may range from psychological morbidity to self-harm (22, 23). The economic costs associated with these conditions are also high. Among doctors and nurses, burnout reduced working hours, and turnover was found to run into thousands of dollars per head (24, 25). In addition to negative outcomes, CARES also demonstrated predictive validity for resilience. When occupational risks are inevitable and potentially a part of the everyday, resilience is a strength indispensable to sustain and grow in the work (26, 27). In the content moderation context, resilience is the psychological capital helping moderators execute complex, ever-evolving workflows as digital content takes on new forms and harms. The rationale for a screening tool like CARES has been to identify candidates who exhibit the potential to benefit from resilience training offered on the job. The current results ratify that indeed CARES holds predictive ability for resilience in the long run. To our knowledge, CARES is the only instrument to assess and predict outcomes unique to content moderators. The tool thus presents itself as a useful screening instrument offering preventative protection to candidates by identifying those with protective traits that can help them cope with the psychological risks of content moderation work.

Furthermore, perceived stress was also significantly predicted by CARES. Increasingly, scholars are highlighting the prevalence of stress in content moderation (17). Strongylou and colleagues (28) observed that among moderators who review “less severe” content such as misinformation and political material, the need to interpret posts based on various nuances due to blind spots in content policy and the resulting increased time spent on resolving these tasks trigger profound stress. Stress holds several implications as it may lead to maladaptive coping—e.g., substance use and other risk behaviors, avoidance—which in the long run negatively impacts wellbeing (29, 30). Early detection of these risks is crucial, and a failure to identify these may lead to graver concerns ranging from psychological morbidity to self-harm (22, 23). Through predicting outcomes, CARES sets the stage to screen for individuals at risk for poor psychological outcomes and to provide a basis for offering adequate psychoeducative wellness programs that will boost longitudinal wellbeing outcomes.

On a more granular level, the REL dimension of CARES was observed to have significant predictive ability in the individual and combined models for all outcomes. Its positive relationship with secondary traumatic stress, perceived stress and burnout, alongside its negative relationship with resilience and compassion satisfaction, reiterates the importance of emotional regulation and flexibility in content moderation work. Petrakaki and Kornelakis (31) explained that emotional labor is the crux of moderators’ workday as they have to manage their own emotions while reviewing different types of content, and also those of users who they may be interacting with as part of moderation processes. To effectively complete their job, moderators must balance emotional engagement with the rational decisions needed in line with policy and regulations. The REL dimension of CARES is particularly useful in determining those at risk for emotional concerns and who may best be considered for alternative professional roles. The PPA and ESc dimensions also demonstrated value especially as seen in their significant independent contributions to compassion satisfaction and resilience. Items in the two dimensions are centered on emotional recovery, empathic outlook, optimism, and social support seeking. Considering the egregiousness of content that moderators may witness firsthand, we turn to research on other frontline workers that has noted the possible development of chronic cynicism, disillusionment and mistrust in others (1, 32) alongside the stigma to solicit help or express one’s psychological concerns to others (33). The characteristics captured in PPA and ESc may aid moderators’ continual faith and confidence in their work and the world at large while also being able to articulate their concerns and benefit from social support. Altogether, the three CARES dimensions captured the vital signs to predict the long-term psychological fit of a candidate for content moderation.

While ESc did not show significant predictive power for the main psychological outcomes analyzed in this study (burnout, stress), it may still capture socially relevant traits that influence other aspects of job or organizational adaptation. For instance, among first responders, the combination of occupational stress and emotional suppression was related to traumatic stress, major depression and generalized anxiety disorders (34). In another study where sociability was experimentally increased, participants reported higher positive affect and life satisfaction (35). The value of ESc might become more evident in future studies involving such variables which were not considered in the current study (limited to broad psychometric screeners). There is a need to explore other outcomes in content moderation roles in order to further explore the relevance of ESc in screening. Additionally, it is worth noting that this analysis was conducted only on participants who went on to be employed. This may have resulted in the exclusion of participants with very low ESc scores, and possibly more negative psychological outcomes. Because of this selection dynamic, some of the potential effects of low ESc might not have been fully captured in this study. Therefore, there is not enough evidence at this stage to justify modifying or removing it from the tool, though further evaluation of its incremental and contextual value is encouraged.

Another valuable finding was the significant negative association of time with secondary traumatic stress. Longer tenured moderators were more likely to experience lower secondary traumatic stress than those with shorter tenure. This contrasts previous literature that cites a positive relationship between tenure and symptom severity among moderators (36, 37). It must nonetheless be noted that the current sample were offered preventative wellness interventions at work which may have helped manage the exposure impact.

## 5 Conclusion

CARES was found to significantly predict all psychological outcomes relevant to content moderators, which supports the predictive validity of CARES. Further, all dimensions showed independent contributions to the different outcomes, underlining the benefit of the 3-factor structure. Lastly, the interaction between CARES dimensions and time across most models was found to be non-significant. However, further research is needed to fully evaluate the impact of time on CARES’ predictive capability.

## 6 Implications

CARES is a preliminary yet necessary step in the direction of systematically quantifying the psychological readiness of candidates seeking content moderation jobs. The three dimensions of the scale cumulatively screen positive and negative competencies with the goal to select right fits while also determining areas for training and skill development. To our awareness, the existing hiring processes for moderators rely on situational tests and behavioral interviews; these may not adequately capture the psychosocial capacities and internal states as efficiently and effectively as a tailored and standardized psychometric tool. Pertinently, by demonstrating predictive validity, CARES provides a robust means to safeguard long-term outcomes of wellbeing and productivity for a workforce with known risks of psychological distress and attrition, for example.

In serving as a preventive and proactive wellness screening tool, CARES sets up opportunities for mitigating adverse outcomes as well as associated repercussions (e.g., health risks, disengaged workforce). By investing in psychometric screening, employees and their organizations benefit from finding their right match not merely from a technical and task perspective but also from a behavioral and value alignment standpoint. CARES helps increase transparency about the need for psychological resilience and coping in jobs like content moderation that require an informed choice to pursue the role.

While the scale’s norms require further validation across settings (we are currently running studies in this regard), we recommend defining categories of acceptance depending on the nature of moderation and specific outcomes of interest. For instance, in teams where highly egregious content is processed, more strict cut-points may be desirable. Additional research is needed to support recommendations for cutpoints in screening, which likely will vary based on the specific use case.

## 7 Strengths, limitations, and future directions

The current study is the first of its kind, to the best of our awareness, that investigated the predictive validity of a tailored employment screener for content moderators. The design included globally validated outcome measures that have strong internal consistency and relevance to outcomes of content moderators, thereby offering a robust assessment of the predictive validity properties of CARES.

However, the study is not without shortcomings. First, the study focused on employees in a single private organization in the Philippines. The exclusion of other companies stems from non-disclosure policies and concerns with data sharing in competitive business environments. This limits the external validity of the results and warrants replication. Future collaborative and multi-site research may involve neutral third-party academic and civil researchers for generalizable findings.

Second, the sample was confined to participants who had consented to match their pre-employment screener scores with their wellness survey data. Candidates who did not provide such consent were excluded during data processing, and may not be fully represented by the current results. Furthermore, pockets of missing data affected the continuity of data and limited analytic choices for maintaining a rigorous design. Both these limitations stem from the voluntary nature of participation and inclusion in analyses which nonetheless is critical to maintain ethics alongside participant trust in the research process.

Another limitation of the current study is the possibility of selection bias due to the sample only consisting of employees who were successfully hired and were still working in the organization at the time of the survey. This limits the current data and excludes those who did not pass the screener during the hiring process and those who left the company during the study period. Therefore, the results must be interpreted within these limits.

A further concern stems from potential bias introduced from the non-normality of outcomes. Most outcomes had skewed distributions such that most reported positive psychological outcomes with fewer reporting moderate or negative outcomes. This resulted in some heteroskedasticity with smaller residuals around the best score (e.g., 10 is the lowest possible score for burnout) resulting from the floor effects. Results of sensitivity analyses showed that transforming the outcome (e.g., log transformation) resulted in the same interpretation with only minor fluctuations in coefficients.

Additionally, despite CARES significantly predicting each of the psychological outcomes, a portion of variance in the random person intercepts, around 71% to 90%, remains unexplained. This suggests that the model may benefit from exploring other factors that may play a role in these psychological outcomes. Future research should explore whether additional predictors may improve prediction as well as whether any grouping variables may moderate these associations. For example, associations between screener and outcomes may be influenced by the individual’s specific role such as whether they are moderating high risk content or are in high volume queues. Although only small amounts of variance were explained across outcomes, this level of prediction may provide capacity for filtering out extreme scores. Future research should explore these associations in a non-restricted sample and set clinically relevant cutpoints to evaluate the screener’s capacity at identifying individuals with significant risk. Additionally, variability was observed within individuals across time. Prospective studies should investigate whether quantifiable workplace factors may explain some of this within person fluctuation (e.g., role changes, management changes, etc.). For instance, workflow issues (e.g., policy changes, inadequate tooling) have been reported elsewhere to induce stress and fatigue in moderators (1, 28). There is a need to evaluate these factors as predictors alongside considering performance and people metrics (KPIs, attrition, absenteeism) as outcomes to enhance the analytical depth and nuances of CARES’ predictive powers. Future research should evaluate what factors may surround the context of the time in which content moderators observe increases and decreases in psychological outcomes from their baseline scores. Studies are also needed to explore whether CARES might predict the extent to which these factors led to changes in psychological outcomes (e.g., CARES predicting magnitude of fluctuations in psychological outcomes).

## Data Availability

The raw data supporting the conclusions of this article will be made available by the authors, without undue reservation.
